# Time Overestimation Devalues Future Rewards: Electroencephalogram Evidence from Intertemporal Choice

**DOI:** 10.3390/brainsci16030271

**Published:** 2026-02-28

**Authors:** Liangliang Yi, Yutong Liu, Haibo Zhou, Chun Lin, Yaru Yang, Xinxin Xiang, Huiyingzi Li, Manling Huang, Xinling Wang

**Affiliations:** 1 School of Education, Hunan University of Science and Technology, Xiangtan 411201, China; 2 Department of Psychology, School of Education Science, Hunan Normal University, Changsha 410081, China

**Keywords:** time perception, intertemporal choice, cognitive control, cognitive resource, impulsive decision-making

## Abstract

**Highlights:**

**What are the main findings?**

- The time overestimation group exhibited greater myopic tendencies in intertemporal decision-making, whereas the time underestimation group was future-oriented.
- Significant differences were observed between the time overestimation and underestimation groups in the N2 and P300 amplitudes.

**What are the implications of the main findings?**

- The research findings indicate that individual cognitive control and cognitive resources play crucial roles in the decision-making process.
- The time overestimation group demonstrated a lower level of cognitive control and fewer cognitive resources, resulting in more impulsive decision-making behavior.

**Abstract:**

**Background/Objectives**: The perceived-time-based model posits that time perception is a critical factor in intertemporal decision-making; however, the mechanisms underlying this influence remain inadequately explored. Despite growing behavioral and neuroimaging findings, no study has directly compared the temporal neural dynamics of individuals who overestimate or underestimate time during intertemporal choices. **Methods**: This study screened participants with time overestimation or underestimation to examine differences in their electroencephalogram (EEG) activity during an intertemporal choice task. **Results**: Behavioral results revealed that the time overestimation group selected the smaller-sooner (SS) option at a higher rate than the time underestimation group, exhibiting a myopic decision-making tendency. EEG results revealed that, compared to the time overestimation group, the time underestimation group exhibited a more pronounced N2 amplitude, an enhanced P300 amplitude, and greater beta band oscillations. Within the time overestimation group, the larger-later (LL) option elicited a more negative N2 amplitude than the SS option. Conversely, in the time underestimation group, the LL option elicited a more positive P300 amplitude than the SS option. **Conclusions**: The results indicate that, during intertemporal decision-making, the time overestimation group experienced more conflict in the LL option, demonstrating lower cognitive control and fewer cognitive resources. This tendency may be driven by a hot system, resulting in more impulsive choices.

## 1. Introduction

Intertemporal choice refers to the process by which individuals weigh costs and benefits across different time points to make judgments and selections [1]. It typically involves choosing between a smaller-sooner (SS) and a larger-later (LL) reward [2]. In everyday life, from daily routines and diets to national policies, intertemporal choices are ubiquitous. They have attracted attention across disciplines. As research progresses, the focus has shifted from descriptive behavioral accounts to in-depth explorations of computational cognitive processes and neural mechanisms [3,4,5]. People often overweight SS options, exhibiting so-called “irrational” myopic preferences [6]. This myopia is often linked to various substance addictions [7,8,9] and impulsivity [10,11,12], whereas a preference for LL options is associated with willpower [11], patience [13], and self-control [10]. Dual-system theory [14] offers a parsimonious explanation for these phenomena. It posits that individuals use two distinct systems to make decisions. The first is the heuristic system (often referred to as System 1), which relies on intuitive and automatic processing, consumes fewer cognitive resources, and tends to lead to more impulsive behavior [15]. The second is the analytic system (System 2), which requires controlled, deliberate processing, consumes more cognitive resources, and favors long-term benefits [16,17,18].

Time perception refers to the subjective experience of objective temporal intervals [19,20]. Takahashi et al. found that time perception follows the Weber-Fechner law, indicating that human intertemporal choices are significantly influenced by the psychophysics of time perception [21]. Building on this, Kim and Zauberman [22] further integrated the relationship between subjective time and intertemporal decision-making, proposing a perceived-time-based model that explains the significant impact of time perception on intertemporal choices [23,24]. Based on this theoretical model, subsequent studies have shown that time-related factors, such as time perspective [25], unpacking [26], and perception strategies [27], can influence intertemporal decision preferences by affecting subjective time. Some factors can indirectly influence intertemporal decisions by affecting time perception, including death awareness [28], episodic foresight [29], incidental affect [30], internet addiction [31], and font color [32]. Simultaneously, individuals often cannot accurately perceive objective time, leading them to overestimate or underestimate actual delays [33,34]. This time perception ability develops across the lifespan, yielding relatively stable trait differences [35,36] and exhibiting notable stability and consistency [23,37,38,39]. Additionally, time perception, as a personality trait factor, appears to be related to personality differences [40]. Previous studies have shown that extraverts are more susceptible to rewards than introverts [41], thus exhibiting a steeper rate of temporal discounting [42]. According to Eysenck’s theory, extraverts tend to perceive time intervals as relatively longer than introverts [43,44], during which they adopt impulsive decision-making strategies and exhibit higher discount rates [42]. However, research exploring the relationship among personality traits, time perception, and intertemporal decision-making from the perspective of cognitive neural mechanisms remains limited.

Previous research has revealed heterogeneity in the influence of time perception on intertemporal decision-making. On the one hand, behavioral studies report that individuals who overestimate time perceive delays as longer, overweight waiting costs, and consequently prefer SS options [33,37,38,45,46]. Neuroimaging studies have identified a high overlap between brain regions involved in time perception and intertemporal decision-making, such as the striatum and the insular cortex [47]. Individuals who underestimate time exhibit enhanced functional connectivity between the parahippocampal cortex and ventromedial prefrontal cortex (vmPFC), enabling them to make future-oriented choices based on subjective time estimates [48]. However, some research findings indicate that time perception does not significantly affect intertemporal decision-making. For instance, Ashare [49] did not find a strong correlation between time perception and impulsive decision-making, whereas Berry [50] discovered no association between time perception and impulsivity. This discrepancy may be attributed to confounding variables, such as temporal priming effects, differences in the duration of waiting periods, emotional states, and other internal and external factors [34,51,52,53]. It may also stem from methodological limitations, particularly the paucity of evidence on the temporal dynamics of decision-making. Previous studies have mostly used behavioral or neuroimaging techniques [37,38,46,54], whereas evidence on temporal dynamics during decision-making is relatively insufficient [47,48]. Given the rapidly changing nature of decision processes, high-temporal-resolution techniques, such as electroencephalography (EEG), can provide more detailed evidence to help clarify these divergent findings.

In the time-domain analysis of EEG research on intertemporal decision-making, P200 is a positive component observed in the early decision stage [55], reflecting attentional processing [18,56]. Conditions that are more attractive to individuals can elicit a larger P200 [57]. Construal Level Theory posits that delayed options have a higher level of abstraction, whereas immediate options are concrete, vivid, and detailed [58], which may lead people to perceive immediate options as more attractive [18,59], thereby exhibiting larger P200 amplitudes. N2, another early ERP component following P200, indexes cognitive control (executive control or executive function [60,61,62]) and conflict monitoring [56,63,64,65,66]. Larger N2 amplitudes indicate stronger cognitive control [18] and correlate positively with the degree of perceived conflict [56]. P300 is a late positive potential that reflects the degree of cognitive resource allocation (including attentional resources) [10,18,63]. Larger P300 amplitudes indicate greater allocation of attentional and cognitive resources [10,67]. During intertemporal decision-making, evaluating the cost–benefit of options requires more attentional and controlled cognitive resources, prompting individuals to make more deliberate decisions [64], which may produce changes in P300 amplitude.

At the time-frequency level of EEG, theta and beta band oscillatory activities are closely associated with decision processes [68]. Previous studies have investigated these frequency bands [65,69,70,71,72]. On the one hand, theta oscillations are associated with conflict processing and cognitive control, reflecting the role of the anterior cingulate cortex in cognitive control [73], and show a positive correlation with conflict [74]. On the other hand, beta oscillations are recognized as an electrophysiological indicator of cognitive processing [12,65,75] and reflect the degree of cognitive resource allocation [76], supporting their use to probe intertemporal decision-making.

In summary, in our study, participants were first screened for individual differences in time perception using a time reproduction task and categorized into time overestimation and time underestimation groups. Thereafter, they performed a classic intertemporal choice task, enabling us to examine behavioral preferences and their neural correlates using time-domain (P200, N2, and P300) and time-frequency (theta and beta) EEG measures, thereby elucidating the temporal dynamics underlying the influence of time perception on intertemporal decision-making.

## 2. Materials and Methods

### 2.1. Participants

Using G-power (3.1.9.7) to estimate the minimum sample size required for this study [77], at a significance level of α = 0.05 and power = 0.8, the simulation results indicated that a sample size of N = 34 was sufficient to detect a medium effect size of f = 0.25. Based on this and referring to previous studies [37,38,45], the present study selected 1050 students enrolled in public computer courses at two universities (Hunan University of Science and Technology and Xiangtan University) to participate in an online time reproduction task (see Experiment 1 for details). All participants’ subjective time estimates were ranked in descending order, and based on the extreme group analysis method [78,79], the top and bottom 27% were selected as the time overestimation and underestimation groups, respectively, consistent with previous studies [80,81,82]. Subsequently, an additional recruitment phase yielded 27 participants from each group who voluntarily agreed to participate in the EEG experiments. During data analysis, individuals with insufficient valid trials were excluded, resulting in a final sample of 25 in the time overestimation group (mean ages = 19.87 ± 1.94 years, 15 females) and 26 in the time underestimation group (mean ages = 19.32 ± 1.59 years, 13 females). All participants in Experiment 2 were right-handed and had no color blindness, normal or corrected-to-normal vision, major illnesses, or psychiatric history. All participants voluntarily participated in the experiment and had not previously participated in similar intertemporal decision-making experiments. Informed consent was obtained before the experiment, and the participants received appropriate compensation after completion. The Ethics Committee of Hunan University of Science and Technology School of Education examined and approved this study in accordance with the Declaration of Helsinki (No. 2025-8).

### 2.2. Experimental Process

First, participants were required to complete a time reproduction task (identical to the online time reproduction task), which was conducted in Experiment 1. The target time intervals to be reproduced included four levels: 2, 4, 8, and 16 s [37,38,45].

Before the intertemporal decision-making task, participants completed a nine-point scale. Referring to the experiment by Suo [37], to minimize potential confounding influences, all participants completed assessments based on nine-point scales across different state dimensions, including hunger level, thirst level, sleep quality, physical state, mental state, anxiety level, emotional state, and patience level, prior to the start of Experiment 2.

Ultimately, in Experiment 2, participants performed the intertemporal decision-making task while an EEG was simultaneously recorded. The formal experiment was conducted in a small electromagnetically shielded room. After wearing the electrode cap, the participants were reminded to focus their attention on the fixation point to avoid excessive eye movement. The stimuli were presented on a 19-inch LCD monitor with participants seated comfortably approximately 65 cm from the screen center, such that the horizontal and vertical viewing angles did not exceed 5°. Experiments 1 and 2 were programmed and data were recorded using lab.js [83] and E-Prime 2.0 software (Psychology Software Tools, Sharpsburg, PA, USA), respectively.

### 2.3. Experimental Tasks and Design

Experiment 2 employed a 2 (time perception: time overestimation vs. underestimation) × 2 (intertemporal options: SS vs. LL) mixed factorial design. Time perception was a between-subjects variable, and intertemporal choice was a within-subjects variable. The dependent variables were the proportion of SS choices in the intertemporal choice task, EEG amplitudes (P200, N2, and P300), and EEG oscillations (theta and beta).

#### 2.3.1. Experiment 1: Time Reproduction Task

**Figure 1:**
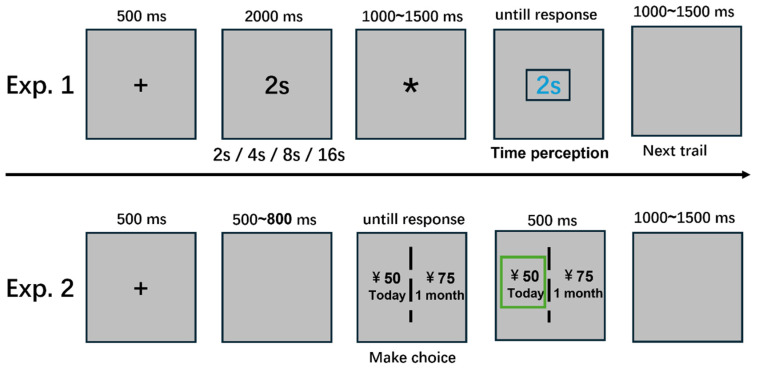
Experiment flowchart. Exp. 1 Time reproduction task. Participants are required to reproduce the previously presented time interval. There are four conditions: 2000 ms, 4000 ms, 8000 ms, and 16,000 ms. For example, if 2000 ms is presented, participants start timing when the blue “2 s” appears. They are required to press the blue number once they subjectively judge that the elapsed time matches the target duration (e.g., 2000 ms), thereby completing the trial. The task included four practice trials and 24 formal trials. Exp. 2 Time course of a single intertemporal choice task. In each trial, two options are presented on the left and right sides of the screen. The positions of the immediate reward and the delayed reward are randomly assigned (left or right) within each trial and balanced throughout the entire experiment. Participants are instructed to respond using the keyboard: press the “F” key to select the left option and the “J” key to select the right option. After the participant’s response, a green frame is placed to indicate the selected response.

The time reproduction task procedure was based on previous studies [20,37,38,45]. As shown in Figure 1, in each trial, a gray screen with a central “+” sign lasting 500 ms alerted participants that the trial was about to begin. Subsequently, a black time number appeared at the center of the screen, for example, “2 s,” indicating the duration of the presented interval (e.g., 2000 ms) and the time to be reproduced in the subsequent task. Next, a black asterisk “*” appeared for a randomly varied duration between 1000 and 1500 ms. Then a blue time number identical to the previous one appeared in the center of the screen. Participants were instructed to start the timing when the blue number appeared and click the blue number when their subjective estimated time matched the duration represented by the blue number, thus completing the time estimation. After 1000–1500 ms, the next trial was initiated.

Before the formal experiment, participants completed practice trials. Tasks with durations of 2, 4, 8, and 16 s were randomly presented once to familiarize participants with the experimental procedures. The formal time reproduction task consisted of 24 trials, with each condition repeated six times and presented in a random order.

#### 2.3.2. Experiment 2: Intertemporal Choice Task

The time and monetary settings of this task were based on previous research [2,56,84,85]. Virtual money was used as the stimulus material. Specifically, a fixed paradigm with randomized reward magnitudes was adopted. Regarding the time settings, the SS option always featured a shorter waiting time labeled “today,” while the LL option involved longer waiting times of “1 month” or “2 months.” The monetary amounts for the SS option were randomly drawn from a Gaussian distribution with a mean of 50 yuan and a standard deviation of 25 yuan and were subsequently converted to integers ranging from 13 to 120 yuan. Meanwhile, the LL option amounts were determined by increasing the SS amounts by 5%, 10%, 15%, 25%, 35%, and 50%. The experiment consisted of four blocks of 80 trials each, totaling 320 trials. Participants completed a practice session (eight trials) before the formal task to ensure that they understood the experimental procedures. Before the experiment, participants were informed that, following the completion of the experiment, the system would randomly select one of their choices to determine the timing of the experimental reward. For example, if the randomly selected choice was “1 month—75 yuan,” the participant would receive the payment after one month.

The procedure for a single trial is illustrated in Figure 1. First, a black fixation point “+” appeared at the center of the screen for 500 ms. This was followed by a randomly timed blank screen lasting 500–800 ms, after which the immediate (SS) and delayed (LL) options were randomly presented on the left and right sides of the screen. Participants were required to indicate their true preference by pressing a key (“F” to select the left option and “J” to select the right option). The options remained on the screen until participants responded. Once a choice was registered, a green frame immediately appeared around the selected option and lasted for 500 ms. This was followed by a blank screen that lasted 1000–1500 ms before the next trial began. The order of the trials and positions of the options (left or right) were randomly assigned. There was a self-controlled rest period of more than 2 min between adjacent blocks.

### 2.4. Data Recording and Analysis

#### 2.4.1. Behavioral Analyses

An independent-samples *t*-test was used to compare the “time overestimation” and “time underestimation group” groups identified in Experiment 1 for the four subjective time interval estimates. In Experiment 2, independent-samples *t*-tests were used to analyze the scores of each item on the nine-point scale and the proportion choosing the SS option to examine the differences between groups.

#### 2.4.2. EEG Recording and Analyses

A 64-channel electrode cap, extended by the international 10–20 system, was used to record EEG signals using Scan 4.5 (NeuroScan Inc., Charlotte, NC, USA) software. During the experiment, an online reference electrode was placed on the left mastoid. During offline preprocessing, the data were re-referenced to the common average across all scalp electrodes [86,87]. Bad channels were identified and interpolated prior to re-referencing to ensure uniform spatial sampling. Moreover, two electrodes were placed on the participants’ right eye sockets (1 cm above and 1 cm below) to record vertical electrooculography (VEOG). Two additional electrodes were placed on the left and right eye areas near the temple to record the horizontal electrooculogram (HEOG). Furthermore, the bandpass filter was set to a range of 0.01–100 Hz, whereas the sampling mode adopted AC sampling (500 Hz sampling rate). All electrode impedances were maintained below 5 kΩ during recording.

EEG data were processed offline using EEGLAB [88]. To eliminate the effects of electrooculogram and movement artifacts, trials were excluded if the signals exceeded ±100 μV. To obtain clean data, blinks, eye movements, electromyography, and other artifact-related components were removed through independent component analysis, which was performed using the EEGLAB toolbox. Based on previous studies, only conditions with more than 25 trials were included for further analysis [2,67,71,89]. The final (mean [M] ± standard deviation [SD]) number of valid trials included in the analysis was as follows: time overestimation-SS condition, 42 ± 8; time overestimation-LL condition, 42 ± 9; time underestimation-SS condition, 43 ± 5; and time underestimation-LL condition, 44 ± 7.

For time-domain analysis, ERPLAB [90] within EEGLAB [88] was used to extract epochs from 200 ms pre-stimulus onset to 1000 ms post-stimulus onset and was baseline corrected using data obtained from −200 ms to 0 ms. For time-frequency analysis, the FieldTrip toolbox [91,92,93] was used to analyze EEG signals from 500 ms pre-stimulus onset to 1500 ms post-stimulus onset. The data were decomposed into frequency bands using a complex Morlet wavelet convolution following the method described by Cohen [94]. Wavelet frequencies ranged from 2 to 40 Hz with 40 frequency bins (linear increase) [95]. The number of cycles for each wavelet was logarithmically spaced between 3 and 10 cycles to achieve a good tradeoff between temporal and frequency precision [96]. The squared magnitudes of these complex signals were recorded at each time point and frequency to acquire the power. Power was then decibel normalized [dB Powertf = 10 × log10(Powertf/Baseline Powerf)], where Baseline Powerf denotes the mean power within a pre-stimulus baseline interval (−500 to −200 ms).

In the time-domain, based on the grand average ERP waveforms and previous studies [2,18,63,64,66,97], the amplitudes of the three time windows (P200, N2, and P300) were analyzed. P200, appearing in the early stage of decision-making [98,99], was measured as the amplitude within the 120–160 ms time window at electrodes F3, Fz, and F4. N2 was measured within the 170–240 ms window at F3, Fz, and F4. P300 was measured within the 250–400 ms window at electrodes P3, Pz, and P4. In the time-frequency domain, consistent with prior research [69,70,71,72], this study focused on the theta (4–8 Hz) and beta (13–30 Hz) frequency bands. Theta power was extracted from electrodes F3, Fz, and F4 during 150–300 ms, and beta power was extracted from electrodes P3, Pz, and P4 during 400–800 ms [73]. A 2 (time perception: time underestimation group vs. time overestimation group) × 2 (intertemporal choice: LL option vs. SS option) mixed analysis of variance (ANOVA) was conducted with time perception as a between-subjects factor. All *p*-values in the ANOVAs were corrected using the Greenhouse–Geisser method.

## 3. Results

### 3.1. Manipulation Check

The results of the independent-samples *t*-test revealed that the time perception scores were consistently higher in the time overestimation group than in the time underestimation group across all four durations. Tables S1 and S2 (Supplementary Materials) present the descriptive statistics and between-group comparison results for the two groups in the online time reproduction task and the time reproduction task, respectively. This consistency confirms that our classification reflects a stable time perception trait (see Supplementary Materials, Tables S3 and S4).

The independent-samples *t*-test indicated no significant differences between the time overestimation and time underestimation groups across various state dimensions (hunger level, thirst level, sleep quality, physical state, mental state, anxiety level, emotional state, and patience level) (all *p* > 0.05); thus, eliminating the influence of these factors on time estimation and subsequent intertemporal choice tendencies (see Supplementary Materials Table S5).

### 3.2. Behavioral Results

**Figure 2:**
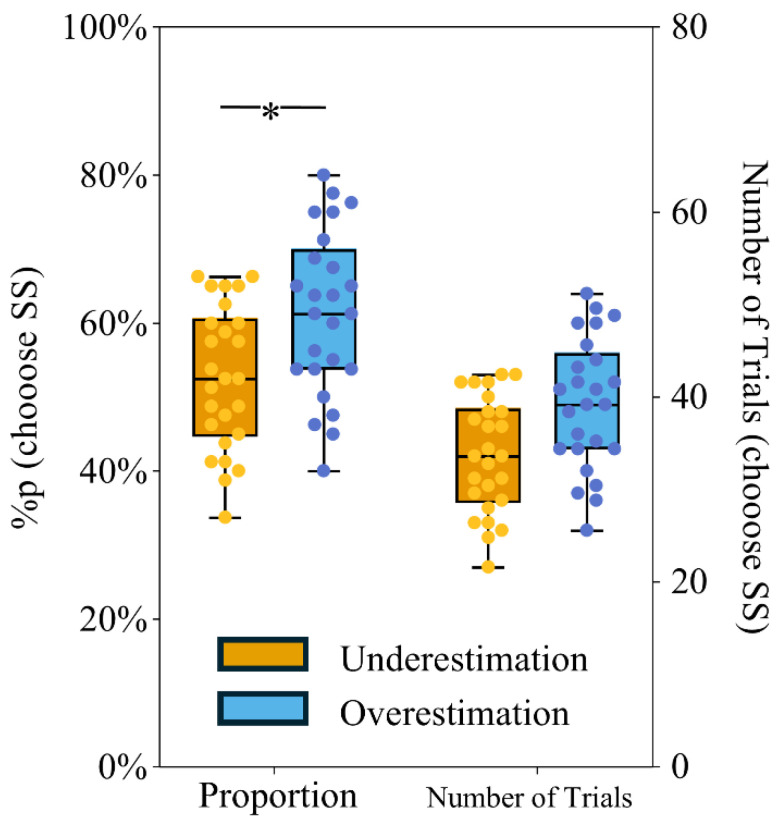
Proportion and number of SS choices. “Proportion” indicates the proportion of choices in which the time underestimation and overestimation groups selected the SS (smaller-sooner) option in the intertemporal choice task. On the right, “Number of Trials” shows the corresponding number of choices. * *p* < 0.05.

The results of the independent-samples *t*-test revealed that the proportion of choosing SS options was significantly higher in the time overestimation group (M ± SD = 0.579 ± 0.084) than in the time underestimation group (M ± SD = 0.512 ± 0.102, *p* = 0.015, 95% confidence interval [CI] = −0.119, −0.014) (Figure 2; Supplementary Materials Table S2).

### 3.3. EEG Results

#### 3.3.1. Time-Domain Results

- P200

**Figure 3:**
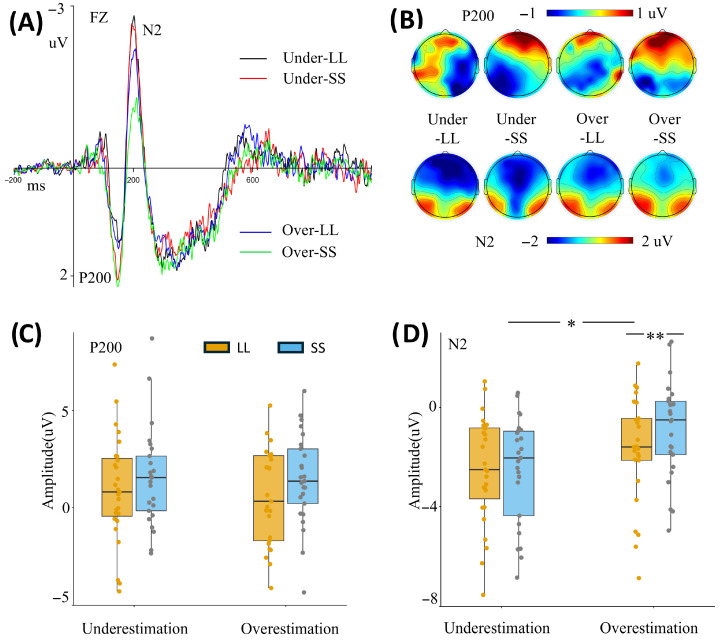
Grand average waveforms (P200 and N2 without additional filtering). Panel (**A**) shows the grand average waveforms at the Fz electrode site. The black lines (Under-LL) represent the time underestimation group–LL condition, red lines indicate the time underestimation group–SS condition, blue lines denote the time overestimation group–LL condition, and green lines show the time overestimation group–SS condition. Panel (**B**) shows the scalp topographies of the P200 and N2. Panel (**C**) shows the average amplitude of P200, and Panel (**D**) shows the average amplitude of N2. “Under” or “underestimation” indicates the time underestimation group, and “Over” or “overestimation” indicates the time overestimation group. LL, larger-later; SS, smaller-sooner. * *p* < 0.05, ** *p* < 0.01.

For the P200 amplitudes, the results showed a significant main effect of intertemporal options [F(1, 49) = 6.021, *p* = 0.018, $\eta_{p}^{2}$ = 0.109]. The SS option elicited a more positive P200 (1.412 ± 0.356 μV) than the LL option (0.751 ± 0.368 μV). No other main effects or interactions were significant (all *p* > 0.05) (Figure 3A; Table 1).

- N2

For the N2 amplitudes, the results showed a significant main effect of intertemporal options [F(1, 49) = 5.771, *p* = 0.020, $\eta_{p}^{2}$ = 0.105]. The amplitude in the LL option (−2.128 ± 0.306 μV) was significantly more negative than in the SS option (−1.677 ± 0.285 μV). The main effect of time perception was also significant [F(1, 49) = 4.592, *p* = 0.037, $\eta_{p}^{2}$ = 0.086], indicating that the N2 amplitude was more negative for the time underestimation group (−2.503 ± 0.392 μV) than for the time overestimation group (−1.302 ± 0.400 μV). The interaction between time perception and intertemporal options was also significant [F(1, 49) = 4.508, *p* = 0.039, $\eta_{p}^{2}$ = 0.084]. The simple effect analysis indicated that in the time overestimation group, the N2 amplitude was significantly more negative in the LL option (−1.727 ± 0.436 μV) than in the SS option [−0.878 ± 0.407 μV; F(1, 49) = 10.044, *p* = 0.003, $\eta_{p}^{2}$ = 0.170] (Figure 3A; Table 1).

- P300

**Figure 4:**
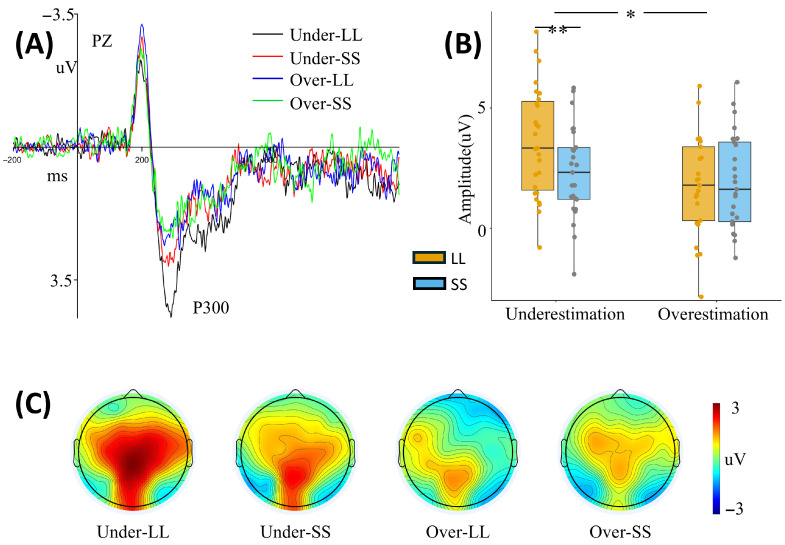
Grand average P300 waveforms (without additional filtering). Panel (**A**) shows the grand average P300 waveform at the Pz electrode site. The black line (Under-LL) represents the time underestimation group–LL condition, the red line represents the time underestimation group–SS condition, the blue line represents the time overestimation group–LL condition, and the green line represents the time overestimation group–SS condition. Panel (**B**) shows the average P300 amplitude, and Panel (**C**) shows the scalp topography of P300. “Under” or “underestimation” indicates the time underestimation group, and “Over” or “overestimation” indicates the time overestimation group. LL, larger-later; SS, smaller-sooner. * *p* < 0.05, ** *p* < 0.01.

For the P300 amplitudes, the results showed a significant main effect of intertemporal options [F(1, 49) = 4.917, *p* = 0.031, $\eta_{p}^{2}$ = 0.091]. The amplitude in the LL option (2.687 ± 0.307 μV) was significantly larger than in the SS option (2.171 ± 0.274 μV). The main effect of time perception was also significant [F(1, 49) = 4.411, *p* = 0.041, $\eta_{p}^{2}$ = 0.083], indicating that the mean P300 amplitude was more positive for the time underestimation group (2.989 ± 0.373 μV) than for the time overestimation group (1.869 ± 0.381 μV). The interaction between time perception and intertemporal options was significant [F(1, 49) = 7.686, *p* = 0.008, $\eta_{p}^{2}$ = 0.136]. The simple effect analysis indicated that in the time underestimation group, the P300 amplitude was significantly larger in the LL option (3.570 ± 0.430 μV) than in the SS option [2.409 ± 0.383 μV; F(1, 49) = 12.698, *p* = 0.001, $\eta_{p}^{2}$ = 0.206]. Meanwhile, the LL option evoked a significantly more positive P300 in the time underestimation group (3.570 ± 0.430 μV) than in the time overestimation group [1.805 ± 0.439 μV; F(1, 49) = 8.251, *p* = 0.006, $\eta_{p}^{2}$ = 0.144] (Figure 4A; Table 1; see Table S6 in the Supplementary Materials for details).

#### 3.3.2. Time-Frequency Results

- Theta band

**Figure 5:**
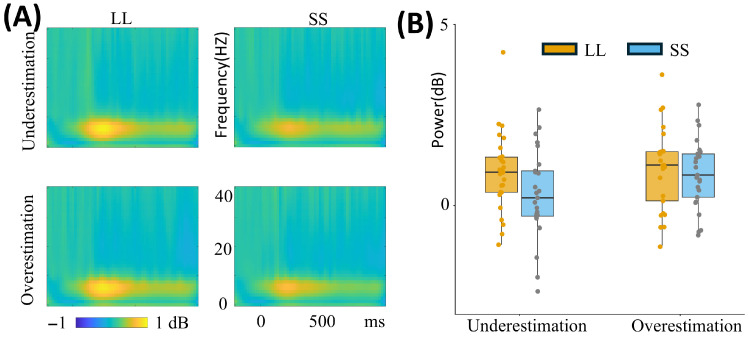
Theta power at the Fz electrode site. Panel (**A**) illustrates theta power at the Fz electrode under four conditions (Underestimation-LL, Underestimation-SS, Overestimation-LL, Overestimation-SS), and the color scales represent normalized power. Panel (**B**) displays the theta power values across these four conditions. “Underestimation” indicates the time underestimation group, and “Overestimation” indicates the time overestimation group. LL, larger-later; SS, smaller-sooner.

For the theta band, the analysis revealed a significant main effect of intertemporal options [F(1, 49) = 5.961, *p* = 0.018, $\eta_{p}^{2}$ = 0.108]. The results indicated that the mean theta power in the LL option (0.981 ± 0.158) was significantly higher than in the SS option (0.587 ± 0.152) (Figure 5A; Table 2).

- Beta band

**Figure 6:**
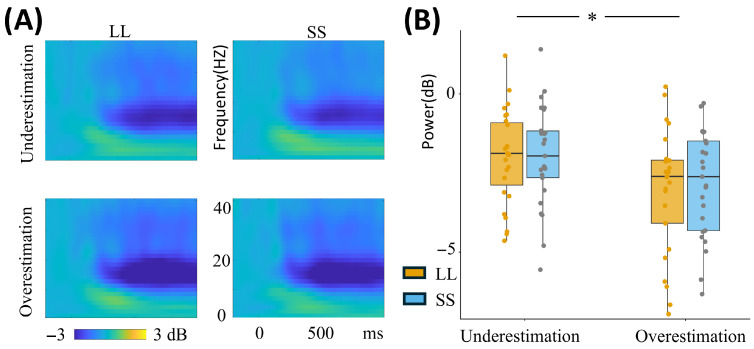
Beta power at the Pz electrode site. Panel (**A**) illustrates beta power at the Pz electrode under four conditions (Underestimation-LL, Underestimation-SS, Overestimation-LL, Overestimation-SS), and the color scales represent normalized power. Panel (**B**) displays the beta power values across these four conditions. “Underestimation” indicates the time underestimation group, and “Overestimation” indicates the time overestimation group. LL, larger-later; SS, smaller-sooner. * *p* < 0.05.

Regarding the beta band, a significant main effect of time perception [F(1, 49) = 4.684, *p* = 0.035, $\eta_{p}^{2}$ = 0.087] was found, with higher mean values of beta power in the time underestimation group (−1.958 ± 0.319) compared to the time overestimation group (−2.946 ± 0.326) (Figure 6A; Table 2; see Table S7 in the Supplementary Materials for details).

## 4. Discussion

Based on the perceived-time-based model, this study examined differences in decision-making tendencies and cognitive processing patterns between time overestimation and time underestimation groups at the behavioral and electrophysiological levels. Behavioral results revealed that a significantly higher proportion of participants in the time overestimation group chose the SS option compared to those in the time underestimation group. Electrophysiological results demonstrated that during the early decision-making stage, the SS condition elicited larger P200 amplitudes, whereas the LL condition elicited more negative N2 amplitudes and higher theta band power. The time underestimation group exhibited more negative deflection N2, whereas the time overestimation group elicited more negative N2 amplitudes in the LL condition than in the SS condition. During the late decision-making stage, the time underestimation group exhibited larger P300 amplitudes and beta power. The LL condition elicited a more positive P300, which was more pronounced in the time underestimation group.

### 4.1. Intertemporal Decision-Making: SS vs. LL

In the early processing stage, SS options elicited significantly greater P200 amplitudes than LL options, which supports the results of previous studies [12]. The P200 reflects stimulus evaluation and rapid judgment [64,100] and is associated with an individual’s attentional level [98]. Larger P200 amplitudes indicate greater attentional allocation to stimuli [56] and faster processing speeds [101]. Previous research indicates that individuals exhibit a relatively stronger preference for SS rewards in intertemporal decision-making [67,102]. Immediate rewards attract more attention, leading to stronger activation of the visual cortex [103]. Thus, we infer that the SS option attracts participants’ attention more significantly during the early processing stage, thereby inducing a larger P200 amplitude [57].

Regarding the N2 amplitude in the time domain, this study revealed that LL options elicited significantly more negative N2 amplitudes than SS options, which also supports previous findings [10,104]. N2 is associated with cognitive control (including response inhibition, response conflict, and error monitoring) [10,18,56,64,66], and its amplitude is positively correlated with conflict intensity [105,106]. In intertemporal decision-making, people generally prefer SS options. Choosing an LL reward requires overcoming this preference, causing individuals to face greater conflict [107], which, in turn, generates a more negative N2 amplitude. In the time-frequency domain, theta oscillations can predict an individual’s preference for the LL option [108], with enhanced theta band activity observed when choosing LL options [109]. The present study supports this view, showing that the LL option elicited greater theta oscillations. More importantly, previous research indicates that the N2 amplitude and theta oscillations may jointly modulate neural activity during cognitive control and conflict monitoring [76,99,110,111,112], indicating that theta power may also reflect the individual’s conflict condition and is positively correlated with the level of conflict [113]. The combined time-domain and time-frequency results of this study suggest that individuals experience greater conflict when faced with the LL option. Furthermore, this study, consistent with previous research [66], indicates that N2 can be regarded as a robust neural marker for measuring an individual’s ability to resist SS temptation in future studies.

During the late processing stage, we observed that LL options elicited significantly larger P300 amplitudes than SS options. This result corroborates previous findings [10,67]. The P300 is associated with cognitive resource allocation, and a larger P300 indicates that more attentional resources are allocated for stimulation [67]. From an evolutionary perspective, future rewards are uncertain [114]. The longer the delay, the greater the risk that the expected or promised reward will not be received [115], which requires people to invest more cognitive resources in evaluating these factors [116,117]. Furthermore, research has found that, compared to choosing SS options, choosing LL options involves decision-making trajectories characterized by prolonged reflection and changes in mind [118], which also leads participants to devote more cognitive and attentional resources [64], manifested as a greater P300 amplitude for LL options.

In summary, during the intertemporal decision-making process, individuals who prefer the LL option typically perform a deeper rational analysis and deliberation, a process that requires more cognitive resources. Conversely, individuals inclined toward the SS option are quickly drawn to immediate rewards early in the decision process, allocate fewer cognitive resources, and therefore display more impulsive behavior. This behavioral pattern provides electrophysiological evidence supporting the dual-system theory [14].

### 4.2. Effects of Temporal Perception

Behavioral data from this study showed that the time overestimation group chose the SS option significantly more often than the time underestimation group. This result supports previous research [37,38,46]. The time overestimation group perceives LL options as occurring further in the future [47], and longer delays are typically associated with increased risk and greater uncertainty [38,119]. Therefore, the time overestimation group subjectively assigns a lower subjective value to delayed rewards, demonstrating a tendency toward more impulsive SS options [37]. The Equate-to-Differentiate Model [120] posits that individuals in the time overestimation group, due to their perception of time as longer, are more inclined to use the time dimension as the basis for intertemporal decision-making [32], thus exhibiting greater myopic tendencies. Simultaneously, these findings are further supported by neuroimaging studies. For example, one study reported that prolonged time perception preferentially activates the medial prefrontal cortex, resulting in a preference for immediate options [121].

Regarding electrophysiological evidence, the time underestimation group elicited a more negative N2 amplitude than the time overestimation group. Previous studies have indicated a close relationship between time perception and cognitive control [122,123,124], with both processes activating brain regions such as the fronto-parietal-insula and putamen [122]. Groups with cognitive control deficits, such as those with internet addiction disorder [125], childhood epilepsy [126], and ADHD [127], often show impaired time estimation abilities. Classic delay-of-gratification studies have also confirmed that individuals who can wait for future rewards generally possess stronger self-control abilities [128,129]. Based on this, we inferred that, in intertemporal choice tasks, the time underestimation group, characterized by a stronger future-oriented preference, likely relies on stronger cognitive control. In the present study, this inference is supported by neuroelectrophysiological data: the time underestimation group suppressed impulsive choices of the SS option through enhanced cognitive control, which was reflected in a more negative N2 amplitude. Conversely, the time overestimation group further supported this inference. Behaviorally, individuals with poorer cognitive control tend to overestimate time [130] and prefer myopic intertemporal choices [38]. Neuroimaging studies have also indicated that individuals who perceive longer time intervals exhibit reduced cognitive control, reflected in preferential activation of the medial prefrontal cortex, which increases impulsivity and promotes SS choices [121].

In early-stage interactions in decision-making, the time overestimation group elicited a more negative N2 amplitude for the LL option than for the SS option. This finding suggests that when the time overestimation group chooses the LL option, they may experience stronger conflict. This conflict arises primarily from the interaction between these two aspects. First, because individuals have a strong preference for immediate options, choosing an LL reward requires overcoming the preference for an SS reward, which increases conflict [107,131,132]. Second, the LL option involves both the uncertainty associated with a longer delay and a higher reward. Individuals who overestimate time perceive greater uncertainty regarding future rewards [133], creating a conflict during the weighing process. Additionally, compared with the preferred option (behaviorally manifested as choices made more frequently), selecting a non-preferred option elicits more negative N2 amplitudes [134]. Based on these findings, we cautiously infer that, since the SS option is the preferred choice for the time overestimation group, choosing the LL option generates greater cognitive conflict, resulting in a larger N2 amplitude.

Regarding late-stage components, this study found that the time underestimation group exhibited a larger P300 amplitude than the time overestimation group. Previous research has found that the time overestimation group exhibited significantly lower cognitive function scores (e.g., attention) than those in the time underestimation group [135]. During the decision-making process, individuals who underestimate time typically invest more cognitive effort, and that time underestimation is closely related to processing a larger amount of information and allocating more cognitive resources [136], thereby eliciting larger P300 amplitudes. Time-frequency analysis revealed that the time underestimation group exhibited stronger beta power than the time overestimation group. This confirms previous findings that participants who underestimated time had greater beta power [137]. Additionally, according to Eysenck’s arousal theory, extraverted individuals tend to have a lower baseline cortical arousal level, which leads them to overestimate objective time [43,44,138]. Consequently, they seek immediate stimulation to increase arousal and have greater difficulty tolerating prolonged periods of low stimulation [139]. The Internal Clock Model posits that time perception depends on a “biological clock” mechanism. Underestimation of objective time is associated with higher arousal levels, during which the internal clock runs faster [140]. This neural mechanism may cause individuals who underestimate time to subjectively perceive waiting periods as shorter, prompting more contemplation and exhibiting more cautious decision-making strategies with lower impulsivity [141]. Therefore, the time underestimation group invests more cognitive resources in prudent deliberation, resulting in more rational decisions and exhibiting larger P300 and beta power.

In the late stage of decision-making, interaction effects were observed: on the one hand, the time underestimation group elicited a larger P300 amplitude for the LL option than the SS option; on the other hand, the time underestimation group showed a greater P300 amplitude for the LL option than the time overestimation group. These interactions indicate that the time underestimation group allocated more cognitive resources to the LL option. Because the LL option entails risk factors, such as high rewards, long delays, and uncertainty [117], individuals need to allocate more cognitive resources to evaluate its costs and benefits [64]. Furthermore, Suo’s research [37] on intertemporal choice tasks found that the time underestimation group tended to make more prudent decisions, whereas the time overestimation group tended to make impulsive choices without thorough deliberation. Consequently, compared with the time overestimation group or the immediately available SS option, the time underestimation group is likely to allocate more attentional and control resources when selecting the delayed LL option, thereby eliciting larger P300 amplitudes [67]. These results consistently indicate that the time underestimation group allocates more cognitive resources to the LL option and engages in more prudent deliberation.

Given the inherent limitations, the conclusions of this study should be interpreted with caution when being generalized beyond the present context. First, this study primarily involved healthy populations, whereas extensive research has focused on clinical populations in separate fields of time perception and intertemporal decision-making. Directly extending these findings to clinical populations (e.g., individuals with autism spectrum disorder, brain injury, or Parkinson’s disease) may be methodologically unwarranted [142]. Furthermore, intake of addictive substances such as drugs, alcohol, or tobacco can temporarily affect an individual’s time perception [143]. Therefore, future research should consider including non-healthy populations or those under the influence of addictive substances to further elucidate the underlying mechanisms. Second, the time reproduction task used seconds as the unit (up to a maximum of 16 s). In everyday life, however, time perception spans a broad range, and short- and long-term intervals may involve different estimation mechanisms [39]. Hence, future studies could incorporate perceptions of different time intervals (e.g., days, months, or years) to explore the influence of time perception on intertemporal decision-making.

## 5. Conclusions

In summary, this study provides electrophysiological evidence supporting the dual-system theory in intertemporal decision-making. The SS option is influenced by heuristic systems that rely on intuitive, automatic processing and consume fewer cognitive resources. In contrast, the LL option is driven by the analytic system, which requires stronger cognitive control and greater cognitive resources. Furthermore, we found that the time overestimation group exhibited relatively lower cognitive control and fewer cognitive resources, making it difficult for them to effectively manage perceived strong conflicts, which behaviorally manifests as more impulsive decisions. In contrast, the time underestimation group demonstrated stronger cognitive control and greater cognitive resource allocation during decision-making, allowing for more prudent deliberation and a stronger tendency toward LL options. This suggests that the hot system (heuristic system) dominates decision-making in the time overestimation group, whereas the time underestimation group is primarily driven by the cool system (analytic system). The differential involvement of these two systems in the decision-making process may jointly shape individuals’ distinct behavioral patterns in intertemporal choices.

## Figures and Tables

**Table 1:** Statistical results of event-related potentials. The table presents the F-values, degrees of freedom (df), *p*-values, and effect sizes ($\eta_{p}^{2}$) for the main effects (time perception, intertemporal options) and interaction effect (time perception × intertemporal options).

		F	df	*p*	$\eta_{p}^{2}$
P200	Time Perception	0.149	1, 49	0.701	0.003
	Intertemporal Options	6.021	1, 49	0.018 *	0.109
	Interaction Effect	0.460	1, 49	0.501	0.009
N2	Time Perception	4.592	1, 49	0.037 *	0.086
	Intertemporal Options	5.771	1, 49	0.020 *	0.105
	Interaction Effect	4.508	1, 49	0.039 *	0.084
P300	Time Perception	4.411	1, 49	0.041 *	0.083
	Intertemporal Options	4.917	1, 49	0.031 *	0.091
	Interaction Effect	7.686	1, 49	0.008 **	0.136

Note: Time underestimation group (N=26); Time overestimation group (N=25). The interaction effect refers to Time Perception × Intertemporal Options. $\eta_{p}^{2}$ = partial eta squared. * *p* < 0.05, ** *p* < 0.01.

**Table 2:** Statistical results of event-related oscillations. The table presents the F-values, degrees of freedom (df), *p*-values, and effect sizes ($\eta_{p}^{2}$) for the main effects (time perception, intertemporal options) and interaction effect (time perception × intertemporal options).

		F	df	*p*	$\eta_{p}^{2}$
Theta	Time Perception	1.521	1, 49	0.223	0.030
	Intertemporal Options	5.961	1, 49	0.018 *	0.108
	Interaction Effect	2.284	1, 49	0.137	0.045
Beta	Time Perception	4.684	1, 49	0.035 *	0.087
	Intertemporal Options	0.927	1, 49	0.340	0.019
	Interaction Effect	0.537	1, 49	0.467	0.011

Note: Time underestimation group (N=26); Time overestimation group (N=25). The interaction effect refers to Time Perception × Intertemporal Options. $\eta_{p}^{2}$ = partial eta squared. * *p* < 0.05.

## Data Availability

Data collection was carried out in strict compliance with the ethical guidelines and regulations of School of Education of Hunan University of Science and Technology. The data presented in this study are available upon request from the corresponding author due to ethical and institutional regulations.

## Supplementary Materials

The following supporting information can be downloaded at https://www.mdpi.com/article/10.3390/brainsci16030271/s1. Table S1: Descriptive Statistics and Difference Analysis of Time Perception Scores (Pre-test); Table S2: Descriptive Statistics and Difference Analysis of Time Perception Scores (Experiment 1, Post-test) and Behavioral Choice Probabilities for Time Overestimation and Underestimation Groups (Experiment 2); Table S3: Descriptive Statistics (M ± SD) of Time Perception (Overestimation/Underestimation) Across Pre-test/Post-test and Different Time Intervals; Table S4: Mixed-Design ANOVA Results for Time Perception Scores by Group (Overestimation/Underestimation), Time (Pre-test/Post-test), and Duration; Table S5: Descriptive Statistics and Difference Analysis of Ratings on Different State Dimensions between the Time Overestimation and Time Underestimation Groups; Table S6: Mean (M) Amplitudes (μV) and Standard Deviations (SD) for P200, N2, and P300 Components; Table S7: Mean (M) Amplitudes (μV) and Standard Deviations (SD) for Theta and Beta Components.

## Abbreviations

The following abbreviations are used in this manuscript:

EEG	Electroencephalography/Electroencephalogram
LL	Larger-Later
SS	Smaller-Sooner
ERP	Event-Related Potential
ERO	Event-Related Oscillation
