# Correction: Yi et al. Time Overestimation Devalues Future Rewards: Electroencephalogram Evidence from Intertemporal Choice. *Brain Sci.* 2026, *16*, 271

**DOI:** 10.3390/brainsci16101119

**Published:** 2026-10-16

**Authors:** Liangliang Yi, Yutong Liu, Haibo Zhou, Chun Lin, Yaru Yang, Xinxin Xiang, Huiyingzi Li, Manling Huang, Xinling Wang

**Affiliations:** 1 School of Education, Hunan University of Science and Technology, Xiangtan 411201, China; 2 Department of Psychology, School of Education Science, Hunan Normal University, Changsha 410081, China

In the original publication [1], the funding information was incorrect. The correct funding information is as follows: Funding was provided by the Philosophy Social Science Project of Hunan Province, Grant No. 20YBQ050 (The Mechanism Underlying the Influence of Scarcity Mindset on Intertemporal Decision-Making in the Context of Relative Poverty) to L.Y. The authors state that the scientific conclusions are unaffected. This correction was approved by the Academic Editor. The original publication has also been updated.
