# Supplementary material for: Time Overestimation Devalues Future Rewards: Electroencephalogram Evidence from Intertemporal Choice
Source: Brain Sci. 2026 Feb 28;16(3):271. doi: 10.3390/brainsci16030271 (PMC13023663; doi:10.3390/brainsci16030271)
Supplement: Supplementary file 1 [file brainsci-16-00271-s001.zip › brainsci-4172993-supplementary.pdf]

Time overestimation devalues future rewards: Electroencephalogram evidence from intertemporal choice

Table S1. Descriptive Statistics and Difference Analysis of Time Perception Scores (Pre-test)

	Time underestimation group	Time overestimation group	t	p	95% CI
	M ± SD (N=26)	M ± SD (N=25)			
2,000 ms	1576 ± 132	2412 ± 219	– 16.575	$< .001^{***}$	[– 936.874, – 734.265]
4,000 ms	3233 ± 299	4376 ± 122	– 17.976	$< .001^{***}$	[– 1271.842, – 1013.299]
8,000 ms	7182 ± 278	8357 ± 111	– 19.991	$< .001^{***}$	[– 1294.893, – 1055.667]
16,000 ms	15135 ± 227	17239 ± 254	– 31.243	$< .001^{***}$	[– 2239.953, – 1969.220]

Note: We reported the means (M) and standard deviations (SD) of the time overestimation group and the time underestimation group under four delay conditions (2, 4, 8, 16 seconds), as well as the statistical indicators (t-value, p-value, and 95% CI) used to test the differences in delay estimation between the two groups. We found significant differences between the two groups across all four delay intervals (p < 0.001), with the time underestimation group's subjective delay estimates being shorter than the objective delays, while the time overestimation group's subjective delays were longer than the objective delays. $^{***}p < 0.001$.

Table S2. Descriptive Statistics and Difference Analysis of Time Perception Scores (Experiment 1, Post-test) and Behavioral Choice Probabilities for Time Overestimation and Underestimation Groups (Experiment 2)

	Time underestimation group	Time overestimation group	t	p	95% CI
	M ± SD (N=26)	M ± SD (N=25)			
2,000 ms	1574 ± 130	2403 ± 209	– 17.089	$< .001^{***}$	[– 926.888, – 731.835]
4,000 ms	3240 ± 335	4362 ± 157	– 15.400	$< .001^{***}$	[– 1269.445, – 973.915]
8,000 ms	7186 ± 357	8388 ± 205	– 14.823	$< .001^{***}$	[– 1366.093, – 1038.314]
16,000 ms	15147 ± 231	17281 ± 307	– 28.101	$< .001^{***}$	[– 2286.261, – 1981.087]
SS %	0.512 ± 0.102	0.579 ± 0.084	– 2.530	0.015^{*}	[– 0.119, – 0.014]

Note: SS% refers to the proportion of choosing the SS (small-sooner) option. **First,** we reported the means (M) and standard deviations (SD) of the time overestimation group

and the time underestimation group under four delay conditions (2, 4, 8, 16 seconds), as well as the statistical indicators (t-value, p-value, and 95% CI) used to test the differences in delay estimation between the two groups. In the post-test, significant differences between the two groups persisted across all four delay intervals ($p < 0.001$), with the time underestimation group's subjective delay estimates being shorter than the objective delays, while the time overestimation group's subjective delays were longer than the objective delays. **Second,** in the last row, we reported the means (M) and standard deviations (SD) of the two groups' choices under the SS (small-sooner) and LL (large-later) conditions, along with the statistical indicators (t-value, p-value, and 95% CI) used to test differences in the probability of choice between groups. The table shows that the time overestimation group chose the SS option more frequently ($p < 0.05$). $^{*}p < 0.05$, $^{***}p < 0.001$.

Table S3**.** Descriptive Statistics (M ± SD) of Time Perception (Overestimation/Underestimation) Across Pre-test/Post-test and Different Time Intervals

Time Perception	Time	2,000 ms	4,000 ms	8,000 ms	16,000 ms
time underestimation	Pre-test	1576 ± 132	3233 ± 299	7182 ± 278	15135 ± 227
	Post-test	1574 ± 130	3240 ± 335	7186 ± 357	15147 ± 231
time overestimation	Pre-test	2412 ± 219	4376 ± 122	8357 ± 111	17239 ± 254
	Post-test	2403 ± 209	4362 ± 157	8388 ± 205	17281 ± 307

Note: M = Mean; SD = Standard Deviation. The pre-test was conducted via an online time reproduction task, while the post-test was performed in the laboratory.

Table S4**.** Mixed-Design ANOVA Results for Time Perception Scores by Group (Overestimation/Underestimation), Time (Pre-test/Post-test), and Duration

	df	F	P	η_p^2
Time Perception	1,49	2222.781	<0.001***	0.978
Time (Pre/Post)	1,49	0.571	0.454	0.012
Duration	3,147	37776.018	<0.001***	0.999
Time Perception × Time	1,49	0.096	0.758	0.002
Time Perception × Duration	3,147	73.235	<0.001***	0.599
Time × Duration	3,147	0.638	0.546	0.013
Time Perception × Time × Duration	3,147	0.389	0.714	0.008

Note. η_p^2 = partial eta squared. Time Perception refers to the grouping factor (time overestimation vs. time underestimation). Time refers to the testing session (pre-test vs. post-test). Duration refers to the four target intervals (2 s, 4 s, 8 s, 16 s).
Specifically, Time Perception was the between-subjects factor, with Time (Pre/Post) and Duration as the within-subjects factors. *** p < 0.001.

Table S5. Descriptive Statistics and Difference Analysis of Ratings on Different State Dimensions Between the Time Overestimation and Time Underestimation Groups

State Dimension	Time underestimation group	Time overestimation group	t	p	95% CI
	M ± SD (N=26)	M ± SD (N=25)			
Hunger Level	5.039 ± 1.483	4.960 ± 1.567	0.184	0.855	[－0.780, 0.937]
Thirst Level	4.962 ± 1.399	4.760 ± 1.234	0.545	0.588	[－0.542, 0.945]
Sleep Quality	4.962 ± 1.399	4.840 ± 1.375	0.313	0.756	[－0.659, 0.903]
Physical State	4.654 ± 1.231	4.800 ± 1.384	－0.399	0.692	[－0.883, 0.590]
Mental State	5.308 ± 1.761	4.840 ± 1.248	1.098	0.278	[－0.390, 1.326]
Anxiety Level	5.000 ± 1.200	5.040 ± 1.207	－0.119	0.906	[－0.717, 0.637]
Emotional State	5.039 ± 1.183	4.640 ± 1.114	1.238	0.222	[－0.248, 1.045]
Patience Level	5.077 ± 1.197	4.960 ± 1.241	0.342	0.733	[－0.569, 0.803]

Note: We reported the means (M) and standard deviations (SD) of the two groups of subjects across eight different state dimensions, as well as the statistical indicators (t-value, p-value, and 95% CI) used to test differences in state dimensions between groups. Independent samples t-test results showed no significant differences between the two groups across the different state dimensions ($p > 0.05$).

Table S6. Mean (M) Amplitudes (μV) and Standard Deviations (SD) for P200, N2, and P300 Components

	Time Perception		Intertemporal Options	
	Time underestimation group	Time overestimation group	LL option	SS option
P200	1.211 (0.471)	0.952 (0.480)	0.751 (0.368)	1.412 (0.356)
N2	– 2.503 (0.392)	– 1.302 (0.400)	– 2.128 (0.306)	– 1.677 (0.285)
P300	2.989 (0.373)	1.869 (0.381)	2.687 (0.307)	2.171 (0.274)

Note: Time underestimation group ($N = 26$); Time overestimation group ($N = 25$). Values are mean amplitudes (μV) averaged across selected electrodes. Standard deviations appear in parentheses.

Table S7. Mean (M) Amplitudes (μV) and Standard Deviations (SD) for Theta and Beta Components

	Time Perception		Intertemporal Options	
	Time underestimation group	Time overestimation group	LL option	SS option
Theta	0.621 (0.185)	0.947 (0.189)	0.981 (0.158)	0.587 (0.152)
Beta	– 1.958 (0.319)	– 2.946 (0.326)	– 2.507 (0.244)	– 2.397 (0.226)

Note: Time underestimation group ($N = 26$); Time overestimation group ($N = 25$). Values are mean amplitudes (μV) averaged across selected electrodes. Standard deviations appear in parentheses.
